# Still Lost in Transition? Perspectives of Ongoing Cancer Survivorship Care Needs from Comprehensive Cancer Control Programs, Survivors, and Health Care Providers

**DOI:** 10.3390/ijerph19053037

**Published:** 2022-03-04

**Authors:** Leslie W. Ross, Julie S. Townsend, Elizabeth A. Rohan

**Affiliations:** Division of Cancer Prevention and Control, National Center for Chronic Disease Prevention and Health Promotion, Centers for Disease Control and Prevention, 4770 Buford Highway, N.E., MS-F76, Atlanta, GA 30333, USA; zmk4@cdc.gov (J.S.T.); irm0@cdc.gov (E.A.R.)

**Keywords:** cancer survivors, comprehensive cancer control program, care coordination, mixed-methods evaluation, public health

## Abstract

Public health agencies have played a critical role in addressing the complex health and mental health needs of cancer survivors. We conducted a mixed-methods evaluation via a Web-based survey (*n* = 51) and focus groups (*n* = 11) with National Comprehensive Cancer Control Program (NCCCP) recipients and interviews (*n* = 9) with survivors, health care providers (HCPs), and patient navigators to explore these audiences’ cancer survivorship information needs and strategies to improve resource dissemination. Participants revealed a need for tailored resources and support for survivors on healthy lifestyle, post-treatment survivorship concerns, psychosocial health, and navigating the health system. HCP needs included education on survivorship care plans and care coordination to facilitate the transition between oncology and primary care. HCPs were survivors’ most trusted source for information; however, participants noted difficulties engaging HCPs in survivorship care. These findings can help public health practitioners focus their efforts to better meet the needs of cancer survivors and their HCPs.

## 1. Introduction

There were more than 16 million cancer survivors in the United States in 2018 [1]. That number is expected to increase, given an aging population and advancements in the early detection and treatment of cancer [2,3,4]. Approximately two-thirds of people with cancer are living 5 years or longer after their diagnosis [2,3]. Although advancements in medical care have drastically improved cancer survival rates, cancer survivors face worse physical and mental health-related quality of life outcomes and a greater risk for additional cancer diagnoses compared with adults without cancer [3,5,6].

Many survivors experience long-term physical, psychosocial, spiritual, and financial challenges after active cancer treatment ends [3,4,7]. Changes to lifestyle factors such as maintaining a healthy weight, adding regular physical activity, tobacco cessation, and routine follow-up and preventive care are all evidence-based strategies that can improve survivors’ quality of life, reduce risk of cancer recurrence, and increase survival [3,8]. However, as a seminal report from The National Academies of Sciences, Engineering, and Medicine (NASEM; formerly the Institute of Medicine) has noted, if the phase between active cancer treatment and post-treatment survivorship care is not carefully coordinated, survivors may become “lost in transition” within the health care system and may lack the knowledge and support needed to maintain a healthy lifestyle and reduce the risk of cancer recurrence [3]. More than one-third of survivors have reported receiving inadequate communication from their health care providers (HCPs) regarding follow-up care, and less than half reported receiving detailed information on late- or long-term effects of cancer treatment, lifestyle recommendations, and psychosocial needs [9]. Use of patient navigation services for cancer survivors is one potential intervention that may increase access to appropriate care. Patient navigators serve as liaisons and advocates for patients across the cancer care continuum and are well positioned to link patients with appropriate resources to reduce barriers to care and improve communication between survivors and their HCPs, especially during pivotal transitions in care [10].

**Figure 1:**
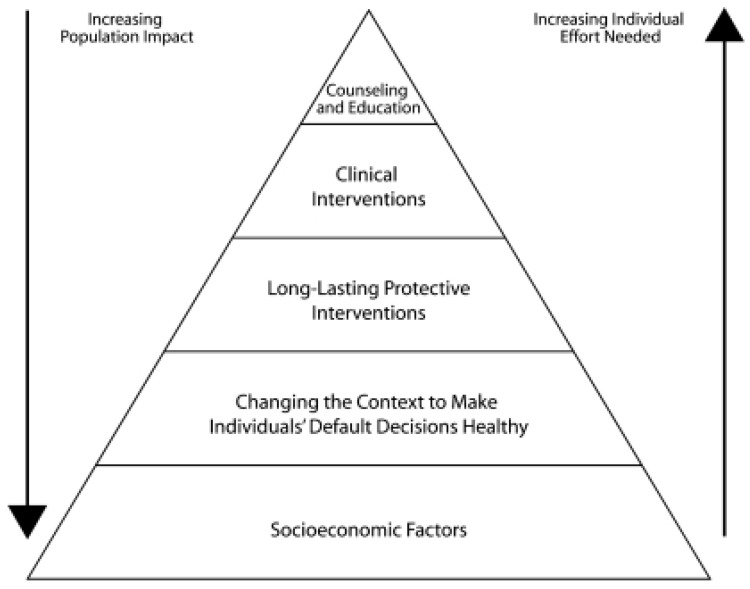
The health impact pyramid. Frieden, T.R. A framework for public health action: The health impact pyramid. Am. J. Public Health 2010, 100, p. 591. Copyright © American Public Health Association 2010 [17].

The Centers for Disease Control and Prevention (CDC) has long played a critical role in strategically addressing the complex health and mental health needs of cancer survivors [11,12,13]. Cancer survivorship is a priority issue for CDC’s Division of Cancer Prevention and Control (DCPC) [12,14]. Through a partnership with LIVESTRONG, DCPC identified priority areas where public health could comprehensively intervene to address survivorship, outlined in the “National Action Plan for Cancer Survivorship: Advancing Public Health Strategies”. The National Action Plan, published in 2004, proposed an approach for addressing survivorship needs within four domains: (1) surveillance and applied research; (2) communication, education, and training; (3) programs, policies, and infrastructure; and (4) access to quality care and services [4]. DCPC’s current survivorship work is primarily accomplished through recipients of DCPC’s National Comprehensive Cancer Control Program (NCCCP) funding, which include all 50 states; the District of Columbia; and select American Indian and Alaska Native tribal organizations, Pacific Island Jurisdictions, and territories [14]. NCCCP recipients work with partners and cancer coalitions to develop and implement tailored action plans across the cancer care continuum, including interventions aimed at improving the quality of life of survivors [12,15,16]. This work is informed by and aligns with the health impact pyramid [17], which depicts public health interventions that have the greatest potential to address social determinants of health and increase population impact on the bottom of the pyramid and interventions that require additional individual-level efforts on the top (see Figure 1). While providing individual-level education and implementing clinical interventions are important, public health practitioners also work with partners to implement evidence-based policy, systems, and environmental (PSE) change strategies that address factors such as socioeconomic status and the built environment that have greater population impact [18,19].

To advance its survivorship work, DCPC funded the establishment of a resource center in 2010 to support the development, implementation, and evaluation of the public health strategies recommended in the National Action Plan [20,21]. The National Cancer Survivorship Resource Center (NCSRC) represented a collaboration between the American Cancer Society, George Washington Cancer Institute, and DCPC, who developed resources, materials, and tools for three primary audiences: (1) cancer survivors and their caregivers; (2) HCPs, including patient navigators; and (3) the policy and advocacy community. Example products from the NCSRC include an information guide to help answer questions that survivors and their caregivers may have about the survivorship journey, clinical follow-up care guidelines and a Web-based training series on survivorship care for primary care providers (PCPs), and a landscape analysis to educate policymakers on priority areas for improving survivorship care [20]. NCCCP recipients and their coalitions collaborated with the NCSRC to create and disseminate relevant, accurate, and evidence-based resources and trainings to the NCSRC’s three audiences.

We conducted a mixed-methods evaluation to determine the extent to which the activities undertaken and materials produced by the NCSRC provided appropriate, evidence- and guidelines-based cancer survivorship messages and met the needs of NCCCP recipients in increasing the implementation of survivorship activities across the various levels of the health impact pyramid. Our analysis for this paper specifically focuses on identifying the ongoing survivorship information and resource needs of survivors and their caregivers, HCPs, and NCCCPs, as well as strategies to improve the reach and dissemination of survivorship resources. The findings from this evaluation were intended to strengthen support provided to cancer survivors by ensuring that current and future needs are met, and to provide the basis for actionable recommendations for improving training and technical assistance for HCPs and NCCCPs related to cancer survivorship. This paper includes results from a quantitative survey that helped identify NCCCP’s awareness of the NCSRC and their perceptions of the information needs of the NSCRC’s audiences, but primarily highlights our qualitative findings, which provided a more nuanced understanding of these needs, as well as how to best reach survivors and HCPs.

## 2. Materials and Methods

We used data collected as part of a comprehensive, concurrent mixed-methods (quantitative and qualitative) evaluation of the NCSRC (OMB No. 0920-0879) [22]. We used a mixed-methods approach to leverage the strengths and mitigate the weaknesses of using quantitative or qualitative methods alone. While a survey can provide a high-level quantification of survivor, HCP, and policy and advocacy community information needs, focus groups and key informant interviews (KIIs) can validate, corroborate, and provide nuance to survey findings. CDC sponsored this evaluation and contracted with Battelle to assist with the design and implementation.

We collected quantitative data from a Web-based survey of NCCCP program directors (PDs) in fall 2016 via SurveyMonkey to identify the information needs of cancer survivors and their caregivers, HCPs, and the policy/advocacy community that PDs believed NCCCPs and their partners should prioritize. The survey also included questions about use and dissemination of specific NCSRC resources that were not a part of our current analysis. We administered the survey to all PDs or their designees in the 65 states, tribal organizations, Pacific Island Jurisdictions, and territories that had received NCCCP funding. We obtained informed consent when survey respondents initiated the survey, and survey completion was voluntary.

After the survey closed, we exported the data from SurveyMonkey into Excel files, converted these files into a de-identified SAS dataset, and cleaned the data. We conducted a descriptive analysis using SAS version 9.3 to obtain frequency counts and percentages among variables of interest [23].

Qualitative data collection consisted of focus groups and KIIs. We recruited focus group participants through the NCCCP recipient survey, selecting NCCCP PDs or their designees who indicated being at least somewhat familiar with the NCSRC resources and having used one or more of them. Thirty potential focus group participants were identified through the survey and then sorted into one of three groups, which focused on one of the NCSRC’s three main audiences: cancer survivors and caregivers, HCPs, and the policy/advocacy community. Participants were sorted into these groups based on which audiences’ materials they had reported using or sharing with partners in the survey. After survey respondents were sorted into the audience groups, we contacted all respondents in each group to determine if they were interested in participating in the focus group interviews. We conducted three 1.5-h focus groups via telephone soon after the NCCCP grantee survey closed in winter 2016. At the beginning of each focus group session, the moderator obtained informed consent and permission to audio record.

To identify potential participants for the KIIs, we asked NCCCP recipients with a strong focus on cancer survivorship to provide referrals to members of their comprehensive cancer control coalitions or other partners that fit in one of the following audience groups: (1) cancer survivors; (2) HCPs, particularly PCPs; and (3) patient navigators. We conducted a total of nine one-hour telephone interviews with the selected audience members between July and November 2016, with three interviewees per audience group. We obtained informed consent and permission to audio record the interviews from the participants at the beginning of each interview.

The focus groups and KIIs were conducted by a two-person team who were trained to implement the evaluation procedures and interview guides: (1) an interviewer with extensive experience in qualitative interviewing; and (2) an assistant who managed the audio recordings and took notes to document the main ideas and key themes in the responses. We de-identified focus group and KII data files (audio recordings and verbatim transcripts) and labeled them according to audience group membership, referring NCCCP recipient, and focus group or KII number. Immediately after each focus group and KII was conducted (or no more than 24 h afterwards), the interview team held a debriefing to identify, discuss, and document the key themes for each question in the interview guides, using the assistant’s notes as a reference. The team compiled key themes from each focus group or KII into preliminary top-line summaries, organized by audience.

We used NVivo 12 Pro software to organize coding and data analysis from the focus group and KII transcripts [24]. To ensure coding consistency, we developed a detailed codebook that included code names, definitions, inclusion and exclusion criteria, rules for when to apply a specific code, and an illustrative quotation example. The codebook contained both deductive codes based on topics from the interview guides, as well as inductive codes that emerged from the data [25]. To ensure consistency, two team members simultaneously coded the first five transcripts in NVivo to compare and agree upon coding practices. Coders kept an audit trail, including process notes and memos detailing coding questions and decisions. We established an iterative process, meeting regularly to discuss nuances of codes, making revisions to the coding and codebook, and identifying emerging codes and themes until we reached a consensus. We conducted a thematic analysis of the data by downloading coding files from NVivo, organizing specific codes based on evaluation questions, and reviewing the text associated with these codes to identify recurring themes and sub-themes [26]. We also compared themes across audience groups to examine if there were thematic differences by group. Once we identified themes, we created a table with detailed descriptions for each theme, including nuances and differences between audience types, as well as relevant quotations from the transcript files.

## 3. Results

### 3.1. Quantitative Survey Results

Of the 65 NCCCP PDs (or their designees) who received the online survey, 51 completed it (78% response rate, which was above our goal of 70%). By jurisdiction, 82% of states and the District of Columbia (*n* = 42), 71% of territories/U.S. Pacific Islands (*n* = 5), and 57% of tribal organizations (*n* = 4) completed the survey.

#### 3.1.1. Awareness of the NCSRC

Fewer than half of the PDs (47%, *n* = 24) were “extremely” or “moderately” familiar with the NCSRC. Of those who were familiar (*n* = 43), nearly half (47%, *n* = 20) first learned about the NCSRC through the CDC, and 72% (*n* = 31) reported sharing these resources with their partners.

#### 3.1.2. Information Needs of Cancer Survivors and Caregivers

**Figure 2:**
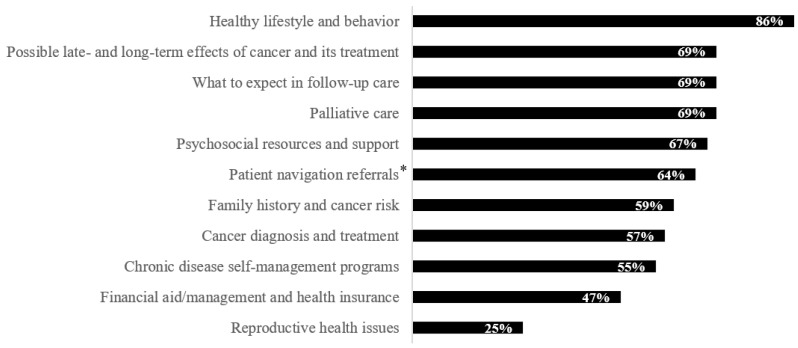
Types of information that program directors reported was “very important” for National Comprehensive Cancer Control Programs and their partners to provide to cancer survivors and their caregivers (*n* = 51). * *n* = 50 for this selection, given that one participant skipped this question.

When asked about the types of information that are “very important” for NCCCPs and their partners to provide to cancer survivors and their caregivers, PDs rated healthy lifestyle and behavior as the most important information need (86%, *n* = 44), followed by possible late- and long-term effects of cancer and its treatment (69%, *n* = 35), what to expect in follow-up care (69%, *n* = 35), palliative care (69%, *n* = 35), psychosocial resources and support (67%, *n* = 34), and patient navigation referrals (64%, *n* = 32) (see Figure 2).

#### 3.1.3. Information Needs of Health Care Providers

When asked about the types of information that are “very important” for NCCCPs and their partners to provide to HCPs regarding cancer survivorship, PDs rated using survivorship care plans as the most important information need (86%, *n* = 44), followed by survivorship care coordination (78%, *n* = 40), prevention and empowering survivors to live well (75%, *n* = 38), clinical follow-up care guidelines for PCPs (63%, *n* = 32), and psychosocial health care needs (61%, *n* = 31).

#### 3.1.4. Information Needs of the Policy and Advocacy Community

PDs reported that it was “very important” for NCCCPs and their partners to provide policy- and decision-makers with survivorship data and statistics (82%, *n* = 42), evidence-based PSE change strategies (80%, *n* = 41), promising practices to promote PSE changes (65%, *n* = 33), and policy briefs (55%, *n* = 28).

### 3.2. Qualitative Focus Group and Key Informant Interview Themes

We held three focus groups with NCCCP recipients (*n* = 11), each of which focused on the needs of a primary audience group of the NCSRC: (1) Cancer survivor and caregiver focus group (*n* = 2; jurisdictions: 1 state, 1 tribal organization); (2) HCP focus group (*n* = 5; jurisdictions: 5 states); and (3) Policy and advocacy community focus group (*n* = 4; jurisdictions: 3 states, 1 tribal organization). We also held nine KIIs with cancer survivors (*n* = 3), HCPs (*n* = 3), and patient navigators (*n* = 3).

We identified key themes within both the focus group and KII discussions, and many themes were corroborated across audience types. These themes included a discussion of the information needs of cancer survivors and their caregivers, HCPs, and NCCCPs, as well as how to improve existing survivorship resources and enhance their reach.

#### 3.2.1. Cancer Survivor and Caregiver Information Needs

Participants within all focus groups and KIIs shared the types of information that they believed was most important to provide to cancer survivors and their caregivers and what new resources and materials would be best suited to address their survivorship needs.

Post-Treatment Survivorship Concerns. Post-treatment survivorship concerns, or remaining concerns following treatment that survivors may have, was a theme that appeared in almost every KII and focus group. Participants spoke about the anxiety survivors experience during the post-treatment period. They may be afraid of cancer recurrence, unsure about what happens after treatment, and confused about the care transition process:

“I’ve had countless calls with patients…it comes almost every time that they finish treatment. They’ll call for that last follow-up and they’re like…I feel so anxious. I’m finally processing all of this and I’m worried about my well-being and recurrence. They get very anxious about recurrence at the end of treatment, because they don’t want to go through what they just went through again.”

—Patient Navigator (KII)

Participants expressed a clear need for up-to-date information and resources to help set appropriate expectations among survivors following cancer treatment regarding who to see and when to go for necessary post-treatment care, side effects they might expect, psychosocial issues they may experience, healthy living tips, and financial concerns. Several participants felt providing survivors with a survivorship care plan, which may contain many of these elements [3,27], was an important way to get them some of this information.

Healthy Lifestyle. A majority of participants spoke about the importance of providing information to survivors on how to build healthy habits to help lower the risk of cancer recurrence. Discussion of healthy lifestyle included a desire for survivorship-specific resources on nutrition, physical activity, oral health, tobacco cessation, and preventive screening. Survivors noted the difficulty in understanding concepts such as how to define healthy eating and knowing how to prepare appropriate healthy food options. They also stated that some survivors may lack the energy or money to prepare healthy meals or incorporate certain types of physical activity:

“Eating healthy. What does that mean? There are so many people that whether they have cancer or not, they don’t eat healthy diets. I don’t know if maybe they don’t understand it or they can’t afford it, or they don’t know how to prepare anything from scratch…A lot of people, they’re not feeling too whippy [energetic] or they’re depressed…and so they eat prepared food and stuff out of a can. That’s hard to address.”

—Survivor (KII)

This survivor also noted the need to consider issues of access in terms of healthy food choices and affordable, safe places to be physically active when recommending healthy lifestyle options to survivors.

Psychosocial Support. Another commonly discussed issue was the need to address survivors’ psychosocial health and provide them with appropriate resources and support. This included help in coping with the grief, depression, and anxiety around being a cancer survivor; changes in sexual function and body image; and the relational shift that some survivors experience in their roles, especially for women who may have been their family’s primary caregiver prior to their diagnosis:

“How is it that I structure my life relationally as a female, a mom, a wife, a grandma? All of my roles have just been up in the air and nobody knows what to do with me. I can’t do the things that I used to do. There is the matter of sexuality. There is the matter of trying to reintegrate in a new way…There are just so many things…The relational quality of life is just left out there.”

—Survivor (KII)

Survivors also spoke about adjusting to a “new normal,” finding meaning after a cancer diagnosis, and how mental health can affect physical health. Many participants felt that helping survivors and their caregivers set expectations and find appropriate mental health services or resources such as support groups could help them process these complicated experiences and emotions.

Navigating the Health System. Several participants mentioned how confusing it can be for patients to navigate the health system and know which HCPs should be part of their care team once cancer treatment has ended. One survivor shared an “anxiety about losing the oncology expertise” during their transition period back into primary care, saying, “One point that was really breathtaking for me was when my oncologist said, ‘I don’t need to see you anymore.’ I’m going what do you mean? You’re my lifeline! What do you mean that you don’t need to see me anymore? In my mind the cancer was still the biggest thing on the screen”.

A focus group participant spoke about the need to educate more survivors that even though their cancer care may be over, they still need regular check-ups and a relationship with a PCP who is aware of the patient’s cancer history. When speaking about this transition in care, a survivor and patient navigator noted the importance of communication between oncology and primary care, which they believed would ideally include a written survivorship plan to convey to the patient who to see for follow-up care and when. A patient navigator also mentioned plans within their program to add a “survivorship navigator” as a resource to help patients navigate their post-cancer treatment care journey.

NCCCP recipients discussed the need to consider health disparities and cultural values when recommending care options. For example, several focus group participants noted that while their health system did have established programs and HCPs available for survivors, many of their patients lived in rural areas and could not access this type of care regularly.

#### 3.2.2. Health Care Provider Information Needs

We also asked HCPs, patient navigators, and the HCP focus group about the types of information HCPs need to provide better care for cancer survivors.

Care Coordination. Related to the previous theme, the most commonly discussed information need for HCPs was how to coordinate and provide better care for cancer survivors. Participants discussed the need to educate HCPs, and in particular, PCPs, on their role in providing care for survivors. Most believed that PCPs should become reengaged in survivors’ care as part of the collaborative care team. However, one HCP believed that a common misconception among PCPs was that survivorship care was the responsibility of the oncologist:

“I think most primary care docs…would say, ‘I think survivorship from a health care standpoint and from delivering health care is the responsibility of oncology and who delivered the cancer care.’ I think that there is going to be first a level of education that might be needed of saying, ‘No, primary care, these patients, once they’re cured from cancer…survivorship [is] actually an area of health care where you’re reengaged.’ I think the reason why there is that strong thought process…[among PCPs is that] when patients get diagnosed with cancer, all of a sudden…we [in primary care] don’t see them anymore. We don’t see them until, all of a sudden, one day they’re on our schedule again.”

—HCP (KII)

Several participants spoke of the need for a better care transition process, including clear communication and collaboration between primary care and oncology to facilitate a “continuum of care” and a “team-based approach.” In the HCP focus group, participants also spoke about the need to provide any other HCPs seeing survivors, such as neurologists, with information on cancer treatment history, including the potential for any long-term side effects, as well as the need to close the gap in referrals to mental health, physical therapy, and rehabilitation services for survivors.

#### 3.2.3. Comprehensive Cancer Control Program Needs

Tailored Materials and Adaptable Resources. A commonly expressed theme in focus groups was the need for tailored survivorship resources for specific communities and for adaptable resources that could be used to insert their own tailored information. Participants reported using some of the NCSRC materials as a great foundation or template for what information to provide. They would then adapt these materials for the appropriate health literacy level, language, age, culture, etc., to make them more accessible to the audiences they served. One participant who worked with American Indian and Alaska Native communities noted that it was not possible for their program to adopt a “one size fits all” approach. To increase engagement, they felt that their materials had to be tailored “down to the tribe”, including photos and stories from “well-known members” of the community. This included a need for tailored materials for HCPs to increase their cultural understanding of these tribal communities, including indigenous medicine practices and beliefs. However, another participant noted that adapting and printing base materials can be expensive, so it would be helpful to have materials that were structured to be easily adaptable, including places to insert their own photos and text.

Data and Information Relevant for their Policy/Advocacy Communities. In the policy focus group, participants spoke about the types of information and data advocates need to educate policymakers in order to work toward more population-wide PSE-level changes. These needs included quality, tailored data as a foundation to justify policy proposals and cost-benefit analyses for budgetary decisions. However, one participant noted that it was also important to add personal stories from survivors and their caregivers in their own voices to help humanize these data, especially for policymakers who “aren’t health-oriented and public health-oriented.” Another participant also wanted information on how to incorporate media advocacy skills to influence decision makers.

#### 3.2.4. Optimizing Survivorship Resources

We also asked participants for feedback on resources available from the NCSRC, including whether they found them to be useful in their practice, what they liked, and how these materials could be improved. Most participants found these resources to be valuable and felt that they were filling a gap in their survivorship needs. While some feedback was very specific to the material it pertained to, we identified several takeaways from participants’ comments that can be applied more generally to future survivorship material development for both survivors and HCPs (see Table 1).

#### 3.2.5. Dissemination of Survivorship Resources: Reaching Survivors and HCPs

To improve reach and dissemination efforts, we asked participants where they typically go to find survivorship information and what they thought the best ways would be for the NCSRC to distribute its resources. See Table 2 for the most commonly discussed feedback regarding improving reach for both survivors and HCPs.

## 4. Discussion

Through triangulation of our quantitative and qualitative data, this evaluation revealed several predominant themes regarding the information and resource needs of cancer survivors and their caregivers, HCPs, and NCCCP recipients, as well as suggestions for how to enhance uptake of existing resources.

Our evaluation found that provision of resources on healthy lifestyle is a significant ongoing need for survivors. A majority of both survey respondents and focus group and KII participants believed that it was important to provide cancer survivors and their caregivers with information on healthy lifestyle and behavior. Past studies have shown that while changes to lifestyle factors have helped to improve cancer survivors’ quality of life and reduce the risk of cancer recurrence, survivors need support to incorporate these healthy lifestyle changes [3,7,8]. In 2018, 32% of adult cancer survivors had obesity, 34% reported no physical activity, and 12% were current smokers [28]. Our survey results also underscored the importance of providing HCPs with information on prevention behaviors and how to empower survivors to live well. Building on this theme, our qualitative analysis indicated that rather than receiving general information about healthy lifestyle behaviors, survivors may need additional details and practical strategies for how to realistically incorporate healthy habits into their daily lives, especially when considering issues related to health disparities, such as whether survivors have access to affordable, healthy food and safe places to be physically active. The types of needs expressed by participants relating to healthy lifestyle reflected not only the upper levels of the health impact pyramid (i.e., those that focus on individual-level counseling and education), but also the lower levels (i.e., the need to address socioeconomic factors and changes to the built environment).

Survey respondents reported that information on possible late- and long-term effects of cancer and its treatment was one of the most important information needs of survivors and their caregivers. This need was corroborated in the focus groups and KIIs; participants spoke extensively about the anxiety survivors experience during the post-treatment period and the fear of cancer recurrence. This finding is consistent with previous literature, which has shown that fear of cancer recurrence is not only common, but can impact the quality of life of cancer survivors [29]. Participants expressed a need for resources—including survivorship care plans—to help survivors set appropriate expectations during the post-treatment period.

Cancer survivors have been shown to have worse mental health-related quality of life and higher rates of depression than adults without cancer [5,6,30]. In addition, most survivors do not report having had a discussion with their HCP about the psychosocial effects of cancer, and thus miss many opportunities to connect with psychosocial services such as counseling or survivor support groups [31]. In our evaluation, a majority of survey respondents believed that it was important to provide both cancer survivors and their caregivers, as well as HCPs, with information on psychosocial resources and support. Focus group and KII participants elaborated on the types of information needed to address survivors’ psychosocial health concerns, which included not only how to cope with common mental health issues such as depression and anxiety, but also how to manage changes in sexual function, body image, and family dynamics.

Most survey respondents believed that it was important to provide survivors and their caregivers with information on what to expect in follow-up care and patient navigation referrals (activities at the top of the health impact pyramid). Focus group and KII participants also spoke about this topic, sharing that survivors need additional resources and support to help them navigate the transition from oncology to primary care. Written survivorship care plans and patient navigation programs were two resources that participants believed could help survivors during this difficult transition period. NASEM recommends that oncology HCPs provide cancer survivors with a comprehensive care summary and follow-up plan (i.e., a survivorship care plan) [3]. Published literature has shown that survivorship care plans can help improve care coordination between oncology and primary care and enhance provider-patient communication during the transition into post-treatment care [20,32,33]. However, fewer than 5% of oncologists have reported providing a written survivorship care plan to their patients [34]. Past studies have also shown that patient navigators can help survivors navigate the health system, facilitate care coordination, promote healthy behaviors, and overcome patient- and system-level barriers to care, such as limited health literacy, which can lead to more equitable outcomes and smoother post-treatment transitions [3,10,32,35,36].

In addition to discussing survivor-specific information needs such as survivorship care plans, focus group participants expressed a need for tailored and adaptable survivorship resources. They believed that a “one size fits all” approach may not work for many communities. Tailoring survivorship resources—including graphics and design—to the culture, language, age group, and other attributes of each intended audience has the potential to increase accessibility and thus use and implementation of survivorship resources. Tailoring to the appropriate health literacy level is particularly prudent, because if survivors have the capacity to understand health information, they are more likely to make informed health decisions and have better health outcomes and decreased rates of hospitalizations [3,32,37]. However, almost half of patients with cancer struggle to understand information about their disease or treatment [38]. A recommendation from attendees of the NASEM Workshop on Health Literacy and Communication Strategies in Oncology was to “strive for clarity and conciseness when communicating complex health information with patients, even if simplification reduces precision” [32]. This principle was reflected by our evaluation participants, who believed that simple, concise survivorship materials would be beneficial for both survivors and HCPs. While receiving survivorship resource materials should not be a substitute for clear, culturally appropriate communication with an HCP, well-designed resource materials can reinforce this information and empower patients and their caregivers to make educated decisions about their health. To ensure appropriate health literacy levels are achieved, it is also important to conduct user testing with members of the intended audience for any new materials developed for survivors [39].

At the clinical level (the next level down from the top of the health impact pyramid), most survey respondents believed that care coordination was an important information need for HCPs, and this was a commonly discussed theme in the HCP focus group and in KIIs with HCPs and patient navigators. Our qualitative findings indicated that PCPs in particular may need additional guidance regarding their role in survivorship care and how to best re-engage survivors in appropriate, evidence-based preventive care management. Past studies have shown that PCPs may lack the formal training and skills needed to provide appropriate follow-up care for cancer survivors, and that additional evaluation could help test models that incorporate PCPs into cancer survivorship care [21,40,41].

NCCCP PDs also expressed a need in both the survey and focus groups for information, data, and survivorship stories to educate policymakers to help enable evidence-based, population-level PSE changes with the greatest potential for high impact (representing the lower levels of the health impact pyramid). Prior literature has shown that provision of dedicated resources to foster PSE change (e.g., addition of a policy analyst, specialized training for program staff and partners, messaging to frame cancer data and strategies) has been instrumental in increasing the capacity of NCCCPs to implement these types of interventions [18,19].

While it is important to identify ongoing needs, many useful survivorship resources already exist, such as those provided by NCCCPs and the NCSRC [11,12,14]; however, these resources cannot help address survivors’ needs if the intended audiences are not aware of them. Even among NCCCP PDs, our survey revealed that fewer than half were at least moderately familiar with the NCSRC. Results from our qualitative analysis revealed several key takeaways on how to improve reach of survivorship resources. Survivors shared that their most trusted source for survivorship information was their HCP. Many survivors may depend on their HCP to identify appropriate resources and translate information depending on their health literacy level. However, participants noted how difficult it can be for HCPs to find time to adequately prioritize and discuss relevant survivorship topics with their patients. Furthermore, as previously noted, research has shown that HCPs, and in particular PCPs, may lack the knowledge and skills needed to provide guidelines-based follow-up care for cancer survivors [3,21,40]. The NCSRC created survivorship guidelines for HCPs on a number of cancer types and developed specific trainings for PCPs [20,42]. This laid the groundwork for DCPC’s development of additional PCP-facing resources to help them address the issues and concerns cancer survivors face [43]. Our analysis indicated that HCPs may also benefit from receiving simplified resources, such as cancer-specific survivorship care checklists, to streamline care when time and competing priorities are a factor.

Several of our participants believed that a collaborative, team-based care approach that includes nurses, social workers, community health workers, patient navigators, and other health care professionals would take the responsibility off individual HCPs to provide education to survivors. This finding reflects a recommendation from NASEM to test models of coordinated, interdisciplinary survivorship care across diverse health systems [3]. While team-based care models have the potential to increase quality improvement and improve health outcomes across the cancer care continuum, future study can identify best practices to better engage various HCPs and educate them on their role in survivorship care coordination, as well as to determine how to best implement these models of survivorship care within both US-based and international contexts [44,45].

NCCCPs and their affiliated cancer coalitions have implemented diverse survivorship initiatives to enhance cancer survivors’ quality of life and address their complex care needs. Through these partnerships, NCCCPs have supported and executed numerous evidence-based interventions relevant to the needs expressed in this evaluation, including programs to help survivors make healthy lifestyle choices, such as eating well, adding physical activity, and quitting smoking; increase the use of survivorship care plans; and connect survivors with mental health support [12]. Programs have also worked to enhance HCP capacity; increase the availability of patient navigation services; build sustainable models of coordinated care; and train HCPs to manage the long-term effects of cancer treatment, screen for recurrence, and provide appropriate preventive care and counseling [12,15]. NCCCPs and their partners are uniquely positioned to continue addressing survivors’ unmet health and mental health needs [13].

### Limitations and Strengths

Our evaluation has several limitations. First, our quantitative survey asked NCCCP PDs their perceptions of the needs of different audiences (survivors and caregivers, HCPs, policymakers), rather than surveying these audiences directly. However, this limitation was offset by hearing directly from survivors and HCPs themselves in the qualitative KIIs. For the qualitative component of the evaluation, our recruitment method of selecting participants who were already familiar with the NCSRC for the focus groups or members of cancer coalitions for the KIIs may have led to a selection bias. While these participants were, in part, selected because they had a broad knowledge base regarding survivorship care needs, their perceptions of priority needs may differ from survivors or HCPs who may be less familiar with this subject area. An additional potential limitation was the small number of focus groups and interviews we were able to conduct, which was due to federal regulation requirements to reduce burden on our participants. While the number of participants we spoke with was relatively low, we did find that we were able to achieve saturation within the three focus groups and nine interviews we conducted, given that common themes were repeated across our quantitative and qualitative data collection methods, and we believed that further data collection would have been redundant [25]. Finally, our study was deeply connected to the specific context of the US health care system. Thus, some of our findings about the health system and cancer care coordination may not be directly relevant for international readers. Still, we believe that many of our findings are relevant for international readers and that further study may be warranted to define cancer survivorship needs within the global context.

A major strength of this evaluation was the use of mixed methods, which can help offset the weaknesses of the quantitative survey or qualitative focus groups and KIIs alone. The triangulation of evaluation results and the inclusion of KIIs with survivors and HCPs helped to corroborate findings from NCCCP PDs [22]. The survey provided a quantification of NCCCP PD’s perceptions about important information needs for survivors, HCPs, and the policy and advocacy community, and the focus groups and KIIs validated these findings and provided nuance and clarity on these themes from the perspectives of both NCCCPs and survivors and HCPs themselves. The focus on the unique perspectives of NCCCPs, who are immersed in the public health and programmatic needs of survivors in their communities, as well as the use of an evidence-based framework (the health impact pyramid) to aid in interpretation of findings, were also strengths.

## 5. Conclusions

For many Americans, cancer has become a chronic condition [2,3]. The public health community and NCCCPs have made great strides in prioritizing and strategically addressing the myriad needs of survivors [11,14,46]. However, further strategic planning can help in reaching the Healthy People 2030 survivorship objectives of increasing the proportion of cancer survivors who live 5 years or longer after diagnosis and increasing quality of life for survivors [47]. NCCCPs can benefit from additional support to provide the resources and education most needed by survivors, caregivers, HCPs, and policymakers to help them make informed decisions [14]. The findings from this evaluation have informed DCPC’s and NCCCP’s efforts to provide the information, resources, and support most needed by cancer survivors and their HCPs and have the potential to help other public health programs prioritize and focus their cancer survivorship efforts. We applied lessons learned from this evaluation to inform a new iteration of a technical assistance and training cooperative agreement with the American Cancer Society and George Washington Cancer Institute to enhance the capacity of NCCCPs to implement cancer survivorship care interventions, as well as a communications contract to update cancer survivorship information and dissemination strategies for DCPC’s website [48]. Improving reach and dissemination strategies can further facilitate access to current information, as well as access to the services, support, and programs needed to improve survivorship outcomes. As the dynamic needs of survivors and their HCPs continue to evolve due to medical advancements and changes to survivorship programs, policies, and guidelines, ongoing study and surveillance is warranted to ensure that public health programs continue to meet survivors’ long-term needs, especially for those who face limited access to quality care and services. Continued study can also ensure that CDC and other public health agencies are providing appropriate, effective, and evidence-based survivorship resources that are reaching the cancer survivors and HCPs who need them most.

## Figures and Tables

**Table 1:** General considerations for enhancing survivorship materials, with illustrative quotes from participants.

	Takeaway	Illustrative Quotes
**Content**	Consider health literacy level of different communities	“Every clinic varies in terms of their socioeconomic profile. A lot of my patients might not be able to read this because they can’t read, and then for others it might be just a little bit a tad too long.”—HCP (KII)
	The simpler and shorter the better, for both survivors and HCPs	“I think that the simpler that you can make it, the better, is my feedback on that just from what I’ve seen with patients. I think just really concise and to the point, efficient, and easy for them to read.”—Patient Navigator (KII) “Those are nice things and purposely keeping it really simple and a checklist and stuff like that. Providers need that more in a sense of because they just have so much going on at any point in care.”—HCP (KII)
**Material & Web Design**	Survivors appreciated relatable quotes/pictures and emotional connection with content	“Oh, my gosh, I felt like somebody understood me when I read some of those quotes.”—Survivor (KII) “I liked that at least three of the major pictures showed a person in a relationship. There was physical touch…That was an effective and emotional connection with this information, because of those pictures and quotes.”—Survivor (KII)
	Online materials should be easy to find and navigate	“There are a lot of good materials that have been developed, but it’s not always easy to remember where. It’s having to remember that resource center, George Washington, or CDC has got tools…You’ve kind of got to remember where the source is in order to find them.”—Policy Focus Group “The more people can hone in on what they’re looking for and find it with as few clicks as possible, the better.”—HCP Focus Group
**Source**	Credibility of the source matters	The American Cancer Society and George Washington Cancer Institute “mean a lot to patients or to providers and so the credibility is really helpful.”—Survivor Focus Group

**Table 2:** Top takeaways on improving reach for survivors and HCPs.

Reaching Survivors	Reaching HCPs
1. Survivors identified HCPs as their first and most trusted source for survivorship information. Survivors also noted that HCPs had limited time to provide survivorship information. One HCP stated patients want their HCPs to spend time explaining and translating information/resources for patients depending on their health literacy level.	1. Focus group participants noted how difficult it can be to connect with HCPs and help them prioritize survivorship. There is “a big hurdle” for HCPs to acquire knowledge about survivorship due to limited time and information overload. Simple resources with checklists that are easy for HCPs to use and implement can help.
2. Many participants said it was common for survivors to search for health information on the Web. Important to point survivors to reputable, accurate survivorship information online. Recommended providing both online and print resources, given some survivors may not be Web-savvy or have access to the Internet.	2. HCPs reported using a variety of channels to access survivorship information. Preferred sources for this information included: ⚬ Conferences and continuing medical education trainings ⚬ Trusted websites (e.g., American Cancer Society, UpToDate) ⚬ Professional organizations ⚬ Printed handouts
3. Several participants noted the importance of timing information and considering the amount of information so as to not overwhelm survivors. Some survivors may not “be ready to think about” survivorship issues until closer to the end of active treatment. Simple, topic-specific resources may help survivors “tackle things one topic at a time.”	3. Several participants highlighted the importance of ensuring that survivorship materials be disseminated to HCPs other than oncologists (e.g., primary care nurses, social workers, cancer support groups, and family services). Recommended a “team-based approach” to providing patient education so it is not the responsibility of only one provider.

## Data Availability

The datasets generated and/or analyzed during the current evaluation are available from the corresponding author on reasonable request.
